# Laparoscopic Spleen-Preserving Distal Pancreatectomy (LSPDP) with Preservation of Splenic Vessels: An Inferior-Posterior Approach

**DOI:** 10.1155/2018/1683719

**Published:** 2018-09-12

**Authors:** Yong Fei Hua, Dipesh Kumar Yadav, Xueli Bai, Tingbo Liang

**Affiliations:** ^1^Department of Hepatobiliary and Pancreatic Surgery, The Second Affiliated Hospital, Zhejiang University School of Medicine, 88 Jiefang Road, Hangzhou, 310009 Zhejiang, China; ^2^Department of Hepatobiliary and Pancreatic Surgery, Ningbo Medical Center Lihuili Eastern Hospital, Medical School of Ningbo University, Ningbo, 315041 Zhejiang, China

## Abstract

**Objective:**

To summarize the operation experience of laparoscopic spleen-preserving distal pancreatectomy (LSPDP) with preservation of splenic vessels by an inferior-posterior dissection of the pancreatic body and evaluate its feasibility.

**Methods:**

Patients undergoing LSPDS at Ningbo Li Huili Hospital and Ningbo Li Huili Eastern Hospital from January 2014 to April 2017 were recruited in this study and were analyzed retrospectively. They were divided into two groups based on the surgical approach: the inferior-posterior approach group and the other approach group. We sought to compare outcomes of the two groups.

**Results:**

The LSPDP procedure was completed successfully in 49 cases, and 48 patients had their splenic artery and vein preserved, including 26 cases in the inferior-posterior approach group and 22 cases in the other approach group. There were no significant differences between the two groups with respect to age (*p* = 0.18), sex (*p* = 0.56), preoperative diabetes (*p* = 1.00), ASA grading (*p* = 1.00), tumor size (*p* = 0.91), intraoperative blood loss (*t* = −0.01, *p* = 0.99), hospital stay (*t* = −0.02, *p* = 0.98), and pancreatic fistula rates (*p* = 1.00). Patients undergoing LSPDP by the inferior-posterior approach had a shorter operative time (*t* = −4.13, *p* < 0.001) than the other approach group.

**Conclusions:**

LSPDS by the inferior-posterior approach associated with shorter operative time is safe and feasible.

## 1. Introduction

With the latest advances in the laparoscopic technique, laparoscopic distal pancreatectomy (LDP) has emerged as a broadly acknowledged surgical technique for a benign or malignant tumor of the pancreas with an advantage of minimally invasive surgery and less postoperative pain [1, 2]. When performing distal pancreatectomy, the spleen is generally removed for easy accessibility, because of its anatomical closeness to the distal pancreas, and for ensuring extensive resection of lymph nodes located along the splenic artery and the splenic hilum. However, a growing concern about the immunological role of the spleen, in conjunction with an inclination toward healthy organ preservation, has led surgeons to avoid splenectomy at some stages during pancreatectomy for benign and low-grade malignant tumors [3]. Moreover, splenectomy may lead to immediate postoperative complications, such as overwhelming postsplenectomy infection (OPSI), subphrenic abscess formation, and hypercoagulability [3]. However, the criticisms of splenic preservation encompass increased operating risk and time and postoperative complications [4]. Nevertheless, preservation of the spleen has been controversial to many; spleen-preserving strategies were associated with similar outcomes to those of splenectomy [5, 6]. Therefore, the patient's quality of life should be taken into consideration whilst deciding on surgical strategies.

In recent years, splenic preservation has increasingly been recommended. Laparoscopic spleen-preserving distal pancreatectomy (LSPDP) has been endorsed as a standard procedure for benign and low-grade malignant tumors in the distal pancreas [7–9].

LSPDP can be completed in either of two methods: (1) carefully isolating the splenic artery and vein from the pancreas via separating and dividing each of the various small branches between the pancreas and these vessels (Kimura's technique) [10] or (2) taking the splenic artery and vein with the pancreas but cautiously preserving the collateral blood supply of the spleen from the short gastric and left gastroepiploic vessels (Warshaw's approach) [11]. Both techniques are accepted for a tumor in the distal pancreas. Obviously, Warshaw's approach is easier. However, it contains a chance of spleen-related morbidity, which includes infarction or abscesses because of inadequate splenic blood supply [2, 11–13]. To minimize this likelihood, the preservation of a sufficient blood supply to the spleen is a priority, and advances in the laparoscopic surgical technique assure the safety of LSPDP with the preservation of the splenic vessels [14]. Different studies have proposed a lateral approach [15] and superior-anterior approach [16] for LSPDP with the preservation of the splenic vessels. However, here, we present our operation experience using an inferior-posterior approach.

We aimed to determine the outcome of LSPDP and compare the inferior-posterior approach with other approaches (lateral and superior-anterior approach). The purpose of this study was to outline our institution's experience, which consisted of 48 patients who underwent LSPDP in our hospital.

## 2. Materials and Methods

### 2.1. Patients

All the patients who underwent LSPDP at the Li Hui Li Hospital and the Ningbo Medical Center, Ningbo, between January 2014 and April 2017 were reviewed retrospectively and were approved by an institutional review board. Data collected from the medical records were age, sex, preoperative diabetes mellitus status, preoperative American Society of Anesthesiologists (ASA) grading [17], pathological diagnosis, operative time, tumor size, intraoperative blood loss, length of hospital stay, postoperative morbidity, and mortality. All patients underwent preoperative CT or enhanced MRI examination to accurately assess the nature of the lesion and its location, size, and relationship with the splenic vessels. The severity of surgical complications was determined according to the Clavien-Dindo classification [18]. Pancreatic fistula was defined according to the guidelines of the International Study Group on Pancreatic Fistulas (ISGPF) [19].

### 2.2. Surgical Procedure and Postoperative Management

**Figure 1:**
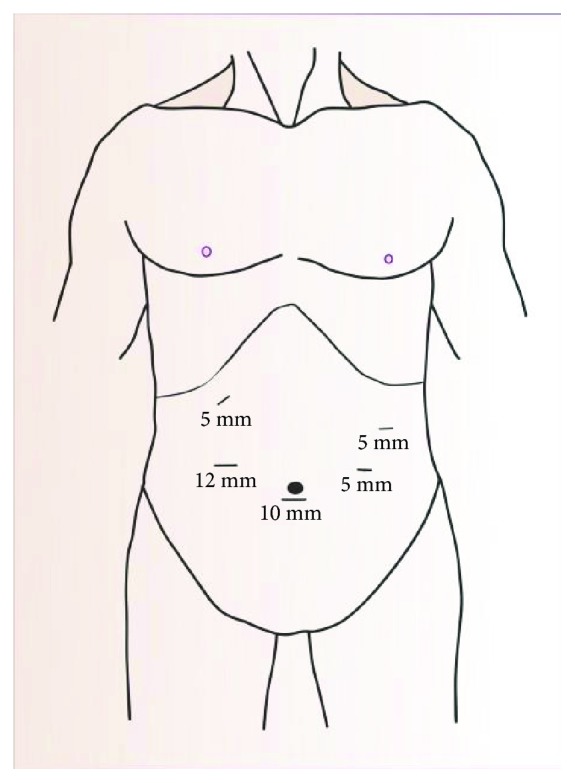
Trocar placement for laparoscopic spleen-preserving distal pancreatectomy (LSPDP).

The patients were placed in the supine position with legs apart on the surgical table and then transferred to reverse Trendelenburg position with the left side elevated. A small incision with a knife was made at the umbilicus for insertion of a 10 mm trocar. Additionally, after the establishment of pneumoperitoneum with a pressure of 13–15 mmHg, a 10 mm trocar was inserted into the umbilicus for the location of a 30° telescope as an observation hole. Then, we further used four trocars under the direct vision of the telescope. Two trocars (12 mm and 5 mm, respectively) were placed in the right upper quadrant for the surgeon, and two 5 mm trocars in the left upper quadrant for the assistant. Port placement is shown in Figure 1.

**Figure 2:**
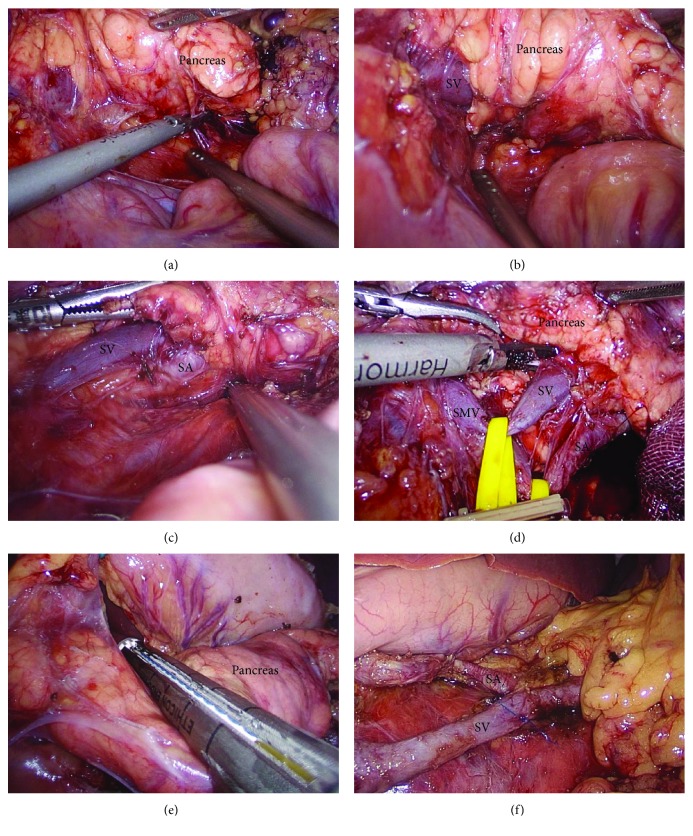
(a) An incision is made in the peritoneum along the inferior border of the body and tail of the pancreas. (b) The body and tail of the pancreas are pulled superiorly and anteriorly. The splenic vein which is embedded in the pancreas is identified. (c) The splenic vein from the pancreas body to the tail was divided, and the splenic artery was exposed. (d) A shortened vessel loop was placed around the splenic vein and artery to provide counter traction and proximal vascular control. (e) The neck of the pancreas is divided with an endoscopic stapler. (f) The splenic vein and artery (the body and tail of the pancreas are removed). SV: splenic vein; SA: splenic artery; SMV: superior mesenteric vein.

The surgical procedure included in inferior-posterior approach LSPDP is as follows:

1. Exploration of the abdominal cavity: we explored the abdominal cavity to exclude puncture damage, metastasis, and other pathological changes in the abdominal organs
2. Exploration and dissection of the pancreas: laparoscopic coagulation shears were used to dissect the gastrocolic and gastrosplenic ligaments further exposing the abdominal surface of the pancreas, and care was taken to preserve the left gastroepiploic vessels and short gastric vessels. The stomach was suspended from the abdominal wall, revealing the pancreas neck, body, and tail. After the exploration of the pancreas, the pancreatic lesion was identified by using intraoperative laparoscopic ultrasound. In addition to this, with the help of laparoscopic coagulation shears, the inferior margin of the pancreas was divided to separate it from the retroperitoneum. Thereafter, the pancreas was then pulled superiorly and anteriorly, further revealing the superior mesenteric vein, the inferior mesenteric vein, and the splenic vein located within the fusion fascia of Toldt. Withal, the longitudinal dissection of the fusion fascia of Toldt toward the tail of the pancreas further revealed the splenic vein which was carefully isolated. Additionally, divulging and isolating the splenic artery were done by gentle traction of the splenic vein caudally using a vascular sling, where the splenic artery lies just above the splenic vein (Figure 2). The dissection then at that point continued from the medial to lateral side, ligating each branch of the splenic vessels encountered supplying the pancreas using laparoscopic coagulation shears or clips. After sufficient surgical margins were attained, the pancreas was transected 2 cm proximal to the tumor using a 45 mm Endo GIA stapler. Further, the pancreatic body and tail were retracted toward the left lateral side, for dorsal side dissection and for freeing the splenic vessels from the distal pancreas by using an ultrasonic knife. To prevent pancreatic fistula, intracorporeal interrupted polypropylene 3-0 sutures were placed on the pancreatic stump. Finally, the specimen was recovered in a bag and pulled out through an extended umbilical port site incision and the specimen was sent for histopathology and was checked for complete hemostasis, and warm water was used to rinse the abdominal cavity; further, a Jackson-Pratt drain (JP drain) was placed near the remnant pancreatic stump on the left side of the subcostal 5 mm port site incision.

Other surgical approaches include the following. The first one is the superior-anterior approach. The patient's position and port placement were as above. In the superior-anterior approach, the splenic artery was identified and isolated first and then dissection of the inferior border of the pancreas is done to separate it from the retroperitoneum further revealing the superior mesenteric vein, the inferior mesenteric vein, and the splenic vein followed by the same step surgical steps as above. The second one is the lateral approach. Dissection of the pancreas from the retroperitoneum and the splenic vessels was commenced from the pancreatic tail and medially toward the pancreatic head. And then the distal pancreas was then retracted medially separating it away from the splenic hilum; further, subsequent surgical steps with the inferior-posterior approach were carried out as described above.

For postoperative management, both groups of patients were managed according to the enhanced recovery after surgery (ERAS) protocol, which especially focused on patients on early mobilization and early nutrition intake [20]. Moreover, at the time of follow-up, Doppler ultrasound was performed to check the patency of splenic vessels.

### 2.3. Definitions

The postoperative complications such as postoperative pancreatic fistula (POPF) [19], postpancreatectomy hemorrhage (PPH) [21], and delayed gastric emptying (DEG) [22] after pancreatic surgery were defined according to the consensus definition of the International Study Group of Pancreatic Surgery (ISGPS).

### 2.4. Statistical Analysis

The analysis was performed using SPSS 19.0 (IBM Corp., Armonk, NY). Continuous data such as age, operative time, blood loss, and postoperative hospital stay are reported as mean ± standard deviation (SD). Categorical data are reported as absolute numbers (*n*). *t*-tests were used to compare continuous variables. Pearson chi-square tests (with Yates' correction) were used to identify differences in categorical variables, and Fisher's exact test was used in the case of a small expected frequency. *p* < 0.05 was considered statistically significant.

## 3. Result

All 49 patients underwent laparoscopic spleen-preserving distal pancreatectomy (LSPDP) without conversion to laparotomy. Among them, 48 patients underwent Kimura's technique and 1 patient underwent Warshaw's technique because of the close proximity of the tumor to the splenic vein. Furthermore, diagnosis was confirmed by routine pathology and immunohistochemistry, including 12 cases of pancreatic mucinous cystadenoma (MCA), 9 cases of pancreatic neuroendocrine tumors (PanNET), 8 cases of solid pseudopapillary tumor (SPT) of the pancreas, 10 cases of pancreatic cyst, 4 cases of intraductal papillary mucinous neoplasm (IPMN), 4 cases of pancreatic serous cystadenoma, and 2 cases of chronic pancreatitis. The four patients in our series with pancreatic serous cystadenoma were only operated when the tumor was larger than 3 cm and the patient was in a state of great anxiety. The mean operation time was 164 ± 40 min, the mean intraoperative blood loss was 136 ± 86 ml, and the mean postoperative hospital stay was 11 ± 4 d. In addition, postoperative pancreatic fistula occurred in 6 cases (32.7%), of which all had grade B pancreatic fistula, and grade C pancreatic fistula did not occur in any of the patients. Cases of pancreatic fistula were managed by drainage tube adjustment, extubation time extension, adequate drainage, and antibiotic therapy. Six patients with grade B pancreatic fistula had abdominal infection, and none of the cases had PPH, DEG, reoperation, and perioperative death. Moreover, postoperative thrombocytosis did not occur in any of the cases; however, splenic infarction occurred in the patients with the spleen preserved after undergoing Warshaw's technique.

Forty-eight cases undergoing Kimura's technique were divided into two groups according to the surgical approach, that is, the inferior-posterior approach group and the other approach group (superior-anterior approach and lateral approach). Patients' demographic characteristics and comparison between the inferior-posterior approach group and other approach group are displayed in Table 1. As described in Table 1, patients in the inferior-posterior approach group had shorter operation time (144.81 ± 27.55 min vs. 186.36 ± 41.75 min, *t* = −4.13, *p* < 0.001) than those in the other approach group, which was statistically significant. There was no statistical difference in age (*t* = −1.37, *p* = 0.18), sex (*p* = 0.56), preoperative diabetes mellitus (*p* = 1.00), preoperative ASA grading (*p* = 1.00), tumor size (*p* = 0.91), intraoperative blood loss (*t* = −0.01, *p* = 0.99), postoperative hospital stay (t = −0. 02, *p* = 0.98), postoperative pancreatic fistula (*p* = 1.00), and grade B pancreatic fistula (*p* = 1.00) between the two groups.

## 4. Discussion

Recently, laparoscopic distal pancreatectomy (LDP) has emerged as the surgical procedure of choice for benign or low-grade malignant tumor of the distal pancreas with advantages of less postoperative pain and early recovery after surgery [1, 2]. Traditionally, the spleen was removed during LDP because of surgical difficulty and its close relationship with the pancreatic tail. However, splenectomy combined with resection of other abdominal organs was found to be associated with high postoperative morbidities such as overwhelming postsplenectomy infection (OPSI), subphrenic abscess formation, hypercoagulability, and even increased risk of cancer [3, 23, 24]. Thus, preservation of the spleen during LDP is recommended.

Laparoscopic spleen-preserving distal pancreatectomy (LSPDP) can be performed by two techniques: (1) Kimura's method [10] and (2) Warshaw's methods [11]. The splenic artery and vein are preserved in Kimura's method, and the normal blood supply of the spleen can be ensured. However, Kimura's technique is difficult and challenging and more susceptible to intraoperative hemorrhage. In contrast, Warshaw's method does not preserve splenic vessels; instead, it preserves the collateral blood supply of the spleen from the short gastric and left gastroepiploic vessels. Technically, Warshaw's method is simple and convenient in comparison to Kimura's method, but splenic infarction remains to be the main complication in Warshaw's method [12, 25]. Therefore, Kimura's technique should be considered first for patients undergoing LSPDP.

Different surgical approaches have their respective advantages and disadvantages for LSPDP with the preservation of the splenic vessels. Most surgeons were accustomed to use the superior-anterior approach [16], because the splenic artery is relatively fixed and it is easy to control the bleeding after the separation and isolation. However, for obese patients and inflamed pancreas, there is a risk of splenic artery injury due to obscurity between the spleen artery and celiac trunk or sometimes due to anatomical variations of the splenic artery. Moreover, some surgeons use the lateral approach [15], where dissection of the pancreas is commenced from the pancreatic tail and medially toward the pancreatic head. This approach does not expose the superior mesenteric vein, and for the tumor near the tail of the pancreas, the free length of the splenic vessels is relatively short; thus, there is a high risk for vessel injury. However, we routinely carry out the dissection from the medial to lateral side in respect to vascular anatomy, for two reasons: (1) in obese patients, the demarcation between the pancreatic tail and splenic hilar is unclear due to excess fat and (2) the splenic hilar region may have anatomical variations of splenic vessels and their branches, which may thus easily lead to injury of the vessels and may subsequently lead to operation difficulty or failure to preserve the spleen.

In spite of the similar outcomes between both the groups in our series of study, operation time was significantly shorter in the inferior-posterior approach group than in the other approach group, probably because surgeons spent more time on the exposure of the splenic artery or stopping the bleeding in some cases of the other approach group. The difference reported here might be caused by the learning curve effect and advancement of equipment to some extent. However, the learning curve is not sufficient to explain why 26 cases on an inferior-posterior approach were done in just 1 year and why only 22 cases on other approaches were previously done over 2 years. And it is still comparatively difficult for us to reveal the splenic artery first. Therefore, we believe that the inferior-posterior approach for LSPDP with preservation of splenic vessels may be more advantageous in revealing and protecting splenic vessels. First, the inferior-posterior approach is consistent with the law of bottom-up laparoscopic view, enabling the operator to perform all operations under direct vision. Secondly, the “fusion fascia of Toldt” was used as the separation plane, which was in accordance with the surgical anatomical features of the pancreas, as it forms an avascular gap between the pancreas and peritoneum, and thus splenic vessels can easily be exposed after its careful dissection. In the embryonic period, the ventral anlage and dorsal anlage of the pancreas rotate counterclockwise, and the membrane of the ventral anlage along with the membrane of the inferior vena cava and abdominal aorta fuses together in the body and tail of the pancreas to form “fusion fascia of Toldt” [26]. In order to free the splenic vessels, our surgical experience suggests that the first clearance from the vascular anatomy by the inferior-posterior approach can reduce risks of bleeding, compared with the other approaches which require the maintenance of a clear operation field. However, a larger series of study is warranted for better evaluation of the assumption.

A few impediments of this study should be recognized. To begin with, the retrospective nature of our series might be subject to biases. Also, a retrospective assessment of the data may have led to differences in characteristics between the two groups. However, the basic characteristics were similar between both the groups as outlined in Table 1. Besides, our study sample is relatively small, and subsequently, interpretation of the results should be done with vigilance. Nonetheless, our study compares two identical groups in a single center. However, LSPDP using the inferior-posterior approach was performed more in recent years. This could prompt a transient favoritism that should be mulled over in the investigation.

In summary, LSPDP using the inferior-posterior approach in combination with the knowledge of the embryonic developmental anatomy of the pancreas can shorten the operation time and does not increase the risk of intraoperative bleeding and postoperative complications. Thus, this approach is feasible and safe, which is worthy of popularization, and can be used for LSPDP whenever reasonable.

## Figures and Tables

**Table 1:** Comparison between the inferior-posterior approach group and other approach group.

	Inferior-posterior approach group (*n* = 26)	Other approach group (*n* = 22)	Statistics	*p* value	*p* value (nonparametric tests)
Age	48.92 ± 16.73	54.86 ± 12.57	−1.37	0.18	0.29
Sex (M/F)	14/12	10/12	0.33	0.56	
Preoperative diabetes					
No	23	19		1.00^∗^	
Yes	3	3			
ASA grading					
Grade I	23	19		1.00^∗^	
Grade II	3	3			
Tumor size (cm)	5.02 ± 2.19	4.96 ± 1.70	0.104	0.91	0.73
Operation time (min)	144.81 ± 27.55	186.36 ± 41.75	−4.13	0.00	<0.001
Intraoperative blood loss (ml)	136.15 ± 89.40	136.35 ± 82.84	−0.01	0.99	0.72
Hospital stay (d)	11.38 ± 3.50	11.41 ± 4.16	−0.02	0.98	0.89
Pancreatic fistula					
No	23	19		1.00^∗^	
Yes	3	3			
Grade B pancreatic fistula					
No	23	19		1.00^∗^	
Yes	3	3			

^∗^Results from Fisher's exact test.

## Data Availability

All the data supporting the results were shown in the paper and are available from the corresponding author upon request.

## References

[1] Mehrabi A., Hafezi M., Arvin J. (2015). A systematic review and meta-analysis of laparoscopic versus open distal pancreatectomy for benign and malignant lesions of the pancreas: it’s time to randomize. *Surgery*.

[2] Melotti G., Butturini G., Piccoli M. (2007). Laparoscopic distal pancreatectomy: results on a consecutive series of 58 patients. *Annals of Surgery*.

[3] Mellemkjoer L., Olsen J. H., Linet M. S., Gridley G., McLaughlin J. K. (1995). Cancer risk after splenectomy. *Cancer*.

[4] Benoist S., Dugue L., Sauvanet A. (1999). Is there a role of preservation of the spleen in distal pancreatectomy?. *Journal of the American College of Surgeons*.

[5] Aldridge M. C., Williamson R. C. (1991). Distal pancreatectomy with and without splenectomy. *The British Journal of Surgery*.

[6] Yamaguchi K., Noshiro H., Yokohata K. (2001). Is there any benefit of preservation of the spleen in distal pancreatectomy?. *International Surgery*.

[7] Nau P., Melvin W. S., Narula V. K., Bloomston P. M., Ellison E. C., Muscarella P. (2009). Laparoscopic distal pancreatectomy with splenic conservation: an operation without increased morbidity. *Gastroenterology Research and Practice*.

[8] Rodríguez J. R., Madanat M. G., Healy B. C., Thayer S. P., Warshaw A. L., Castillo C. F.-D. (2007). Distal pancreatectomy with splenic preservation revisited. *Surgery*.

[9] Warshaw A. L. (2010). Distal pancreatectomy with preservation of the spleen. *Journal of Hepato-Biliary-Pancreatic Sciences*.

[10] Kimura W., Yano M., Sugawara S. (2010). Spleen-preserving distal pancreatectomy with conservation of the splenic artery and vein: techniques and its significance. *Journal of Hepato-Biliary-Pancreatic Sciences*.

[11] Warshaw A. L. (1988). Conservation of the spleen with distal pancreatectomy. *Archives of surgery*.

[12] Jain G., Chakravartty S., Patel A. G. (2013). Spleen-preserving distal pancreatectomy with and without splenic vessel ligation: a systematic review. *HPB*.

[13] Jean-Philippe A., Alexandre J., Christophe L. (2013). Laparoscopic spleen-preserving distal pancreatectomy: splenic vessel preservation compared with the Warshaw technique. *JAMA Surgery*.

[14] Hwang H. K., Chung Y. E., Kim K. A., Kang C. M., Lee W. J. (2012). Revisiting vascular patency after spleen-preserving laparoscopic distal pancreatectomy with conservation of splenic vessels. *Surgical Endoscopy*.

[15] Nakamura M., Nagayoshi Y., Kono H. (2011). Lateral approach for laparoscopic splenic vessel-preserving distal pancreatectomy. *Surgery*.

[16] Inoko K., Ebihara Y., Sakamoto K. (2015). Strategic approach to the splenic artery in laparoscopic spleen-preserving distal pancreatectomy. *Surgical Laparoscopy, Endoscopy & Percutaneous Techniques*.

[17] Doyle D. J. G. E. (2018). American Society of Anesthesiologists classification (ASA class). *StatPearls*.

[18] Clavien P. A., Barkun J., de Oliveira M. L. (2009). The Clavien-Dindo classification of surgical complications: five-year experience. *Annals of Surgery*.

[19] Bassi C., Marchegiani G., Dervenis C. (2017). The 2016 update of the International Study Group (ISGPS) definition and grading of postoperative pancreatic fistula: 11 years after. *Surgery*.

[20] Joliat G. R., Labgaa I., Petermann D. (2015). Cost-benefit analysis of an enhanced recovery protocol for pancreaticoduodenectomy. *The British Journal of Surgery*.

[21] Duarte Garcés A. A., Andrianello S., Marchegiani G. (2018). Reappraisal of post-pancreatectomy hemorrhage (PPH) classifications: do we need to redefine grades A and B?. *HPB*.

[22] Malleo G., Crippa S., Butturini G. (2010). Delayed gastric emptying after pylorus-preserving pancreaticoduodenectomy: validation of International Study Group of Pancreatic Surgery classification and analysis of risk factors. *HPB*.

[23] Kristinsson S. Y., Gridley G., Hoover R. N., Check D., Landgren O. (2014). Long-term risks after splenectomy among 8,149 cancer-free American veterans: a cohort study with up to 27 years follow-up. *Haematologica*.

[24] McGory M. L., Zingmond D. S., Sekeris E., Ko C. Y. (2007). The significance of inadvertent splenectomy during colorectal cancer resection. *Archives of surgery*.

[25] Partelli S., Cirocchi R., Randolph J., Parisi A., Coratti A., Falconi M. (2016). A systematic review and meta-analysis of spleen-preserving distal pancreatectomy with preservation or ligation of the splenic artery and vein. *The surgeon*.

[26] Kimura W., Moriya T., Ma J. (2007). Spleen-preserving distal pancreatectomy with conservation of the splenic artery and vein. *World journal of gastroenterology*.

